# DCTPep, the data of cancer therapy peptides

**DOI:** 10.1038/s41597-024-03388-9

**Published:** 2024-05-25

**Authors:** Xin Sun, Yanchao Liu, Tianyue Ma, Ning Zhu, Xingzhen Lao, Heng Zheng

**Affiliations:** School of Life Science and Technology, China Pharmaceutical University, 24 Tongjiaxiang, Nanjing, 210009 P. R. China

**Keywords:** Databases, Drug discovery

## Abstract

With the discovery of the therapeutic activity of peptides, they have emerged as a promising class of anti-cancer agents due to their specific targeting, low toxicity, and potential for high selectivity. In particular, as peptide-drug conjugates enter clinical, the coupling of targeted peptides with traditional chemotherapy drugs or cytotoxic agents will become a new direction in cancer treatment. To facilitate the drug development of cancer therapy peptides, we have constructed DCTPep, a novel, open, and comprehensive database for cancer therapy peptides. In addition to traditional anticancer peptides (ACPs), the peptide library also includes peptides related to cancer therapy. These data were collected manually from published research articles, patents, and other protein or peptide databases. Data on drug library include clinically investigated and/or approved peptide drugs related to cancer therapy, which mainly come from the portal websites of drug regulatory authorities and organisations in different countries and regions. DCTPep has a total of 6214 entries, we believe that DCTPep will contribute to the design and screening of future cancer therapy peptides.

## Background & Summary

Cancer is a leading cause of death and a significant barrier to increasing life expectancy worldwide^1^. Cancer treatment has improved in the past few decades, but chemotherapy remains the mainstay of cancer treatment. Multidrug resistance is a major problem associated with anticancer chemotherapy^2,3^. Data show that 90% of cancer deaths can be attributed to multidrug resistance^4^. Due to structural differences with small-molecule compounds, bioactive peptides have received much attention and are believed to be alternative candidates for multidrug-resistant cancer therapy^5–7^.

Anticancer peptides (ACPs) are biologically active peptides with antitumor activities that exist widely in a variety of organisms, including mammals, amphibians, insects, plants, and microorganisms. ACPs have many advantages in the treatment of tumours, such as low molecular weight (compared to protein-based therapy), simple structure, high anticancer activity, high selectivity, fewer side effects, easy modifications, and less possibility to cause resistance^8,9^. Although ACPs have been extensively studied, its mechanism of action is not fully understood. At present, the known mechanisms of ACPs mainly include inhibition of tumour cell proliferation or migration^10,11^, inhibition of tumour blood vessel formation^12^, causing cancer cell lysis^13^, and induction of cancer cell apoptosis^14^. In addition, peptides can also serve as a targeted therapeutic agent that can target and directly bind specific cancer cells or cancer related biomarkers, and can also serve as a peptide carrier linked to traditional anticancer drugs^15,16^.

Although the enormous potential of peptides in cancer therapeutics, there is a relative scarcity of dedicated databases specifically storing cancer therapy peptides information. Most of the ACPs information is dispersed in bioactive peptide databases, such as DRAMP^17^, APD^18^, DBAASP^19^, HORDB^20^, CPPsite^21^, and SATPdb^22^, which mainly focus on antimicrobial peptides or hormones. The CancerPPD^23^ database is a known database for annotating ACPs and anticancer proteins; however, its data have not been updated since 2015. Many antimicrobial peptide databases also store information about the anticancer activity of some antimicrobial peptides, but it does not contain detail annotation of ACPs. For example, they did not fully provide information on cancer cells or molecular targets of ACPs, nor do they include peptide drugs. Therefore, we constructed an open, comprehensive database of cancer therapy peptides, DCTPep, that not only includes traditional ACPs, but also peptides with targeted effects on cancer therapeutics. DCTPep can be freely accessed and downloaded from http://dctpep.cpu-bioinfor.org/.

Developing targeted therapies that selectively act on cancer cells has always been an ideal approach for cancer treatment. A promising targeted therapy is drug conjugates, which involve linking targeting carriers with chemotherapy drugs or cytotoxic agents through a linker, such as antibody-drug conjugates (ADCs) and peptide-drug conjugates (PDCs)^24^. Currently, the most common drug conjugates used in cancer treatment in clinical practice are ADCs. However, with the increasing presence of peptides in clinical, PDCs has also emerged. PDCs have the potential to overcome the limitations of ADCs, such as smaller molecular weight and ease of synthesis^25^. Nowadays, only two PDCs, ^177^ Lu-dotatate (DCTPepD0013) and Melflufen (DCTPepD0108), have been approved for clinical cancer treatment, of which Melflufen being withdrawn from the market by the FDA. However, there are still many PDCs in cancer clinical development or about to enter clinical trials. The potential of PDCs cannot be ignored. Peptides play a crucial role as carriers in PDCs. Therefore, DCTPep not only focuses on collecting ACPs but also emphasizes the collection of cancer targeted peptides. The carrier peptides in PDCs include cell-penetrating peptides (CPP) and cell-targeting peptides (CTP)^26^. The classification field in the database also follows a similar category, including cell-penetrating peptides, cancer-targeting peptides, and targeted peptide conjugates.

**Fig. 1:**
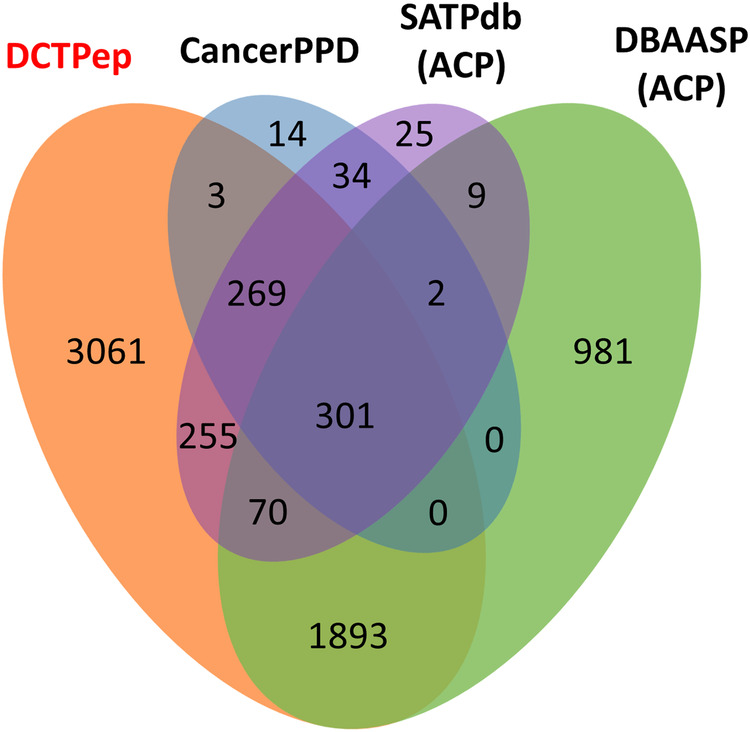
Venn diagram illustrating the numbers of overlapping and non-overlapping peptide sequences related to cancer therapy from the DCTPep, CancerPPD, SATPdb and DBAASP.

Figure 1 and Table 1 presents the comparative results of DCTPep datasets with ACP datasets in other peptide databases. Compared to DBAASP, CancerPPD and SATPdb, DCTPep possesses over 3000 unique entries. DCTPep provides a vast amount of cancer therapy peptide data, including clinically relevant peptide drugs curated in the drug library, filling the gaps in existing data and offering assistance in the design and screening of novel cancer therapeutic peptides. Particularly, the targeted peptide data will offer more options for PDC design. In order to better understand the mechanism of action of cancer therapy peptides, we have added target annotations and collected over 60 targets for these peptides that are not included in other ACPs databases. The dataset is freely available to all via the web without the need to login or registration and is not password protected. We believe that DCTPep will become a valuable resource for the development of novel bioactive peptides, particularly in the field of cancer therapeutics.

**Table 1:** Comparison of peptides related to cancer therapy in DCTPep with other peptide databases (data as of 2023.12.20).

Features	DCTPep	CancerPPD	DRAMP 3.0	APD3	DBAASP v3	SATPdb
Entries related to cancer therapy peptide	6106	624	455	276	3,409	1,099
ACP drugs	108	NA	NA	NA	NA	NA
Cancer cell line	>400	249	NA	NA	NA	NA
Targets	>60	NA	NA	NA	NA	NA
predicted structures of ACPs	1646 (Based on AlphaFold)	617 (Based on molecular dynamics)	NA	NA	1372 (Based on molecular dynamics)	NA
Download dataset	Yes	Yes	Yes	Yes	Yes	Yes

## Methods

### Data collection and compilation

In order to develop DCTPep, extensive searches were conducted on published articles, patents, and public databases. The data of DCTPep was stored in two sub libraries: peptide library and drug library. The inclusion criteria for the peptide library in the DCTPep were as follows: 1. The sequence of amino acids is reported; 2. Mature peptide sequences without precursor and signal regions; 3. The length of the sequence does not exceed 100 amino acids; 4. Peptides that exhibit anticancer/antitumor activity or target specific molecules/biomarkers overexpressed in cancer cells; 5. Cell-penetrating peptides that can enhance the delivery of drugs into cancer cells. The inclusion criteria for drug library were similar to those for peptide library: 1. Peptides and their derivatives or amino acid derivatives related to cancer treatment; 2. Entered clinical research or approved by FDA, EMA or HC.

To collect peptide data, keywords were used to search in academic search engines such as Google Scholar, Web of Science, PubMed, and Google Patents. The keywords included “ACP”, “antiangiogenic peptides”, “cancer therapy peptide”, “cancer targeted peptide”, and “peptide conjugates”. After collecting research papers, patents, and clinical research literature, data were manually extracted. In addition to manually extracting information of cancer therapy peptide from literature, also included other information related to peptides (such as three-dimensional structures) in UniProt^27^, PDB^28^, and other databases. The physicochemical information of peptides is calculated using Expasy Protparam server (https://web.expasy.org/protparam/, accessed on March 2024) and SciDBMaker^29^.

The data of drug library mainly originated from the portal websites of drug regulatory authorities and organisations in several countries and regions. In addition, it was supplemented by the drug databases DrugBank^30^, PubChem^31^, NCI Thesaurus^32^ and Global Substance Registration System (GSRS)^33^. By entering keywords such as “peptides and their derivatives”, “amino acids and their derivatives”, and “anticancer” into the aforementioned website or database, relevant information can be found.

### Structural prediction and evaluation

Due to the difficulties in experimental determination of peptide and protein structures, most of the peptides lack experimental determined structures. AlphaFold^34^ was used to predict the potential 3D structures of DCTPep peptides. Default structure parameters for AlphaFold prediction were used: peptide was modeled as a monomer; Multiple sequence alignment (MSA) information databases: full_dbs (all gene databases)^34^. Each peptide generates 5 structures, and the structure with the highest score is selected based on predicted local distance difference test (pLDDT)^34^. To evaluate the reliability of AlphaFold predicted peptide structures, 30 peptides with experimental determined structures were selected and their structures were predicted by AlphaFold. The differences between predicted structure and experimentally determined structure were calculated by Root-Mean-Square Deviation (RMSD)^35^. Given two conformations, α and β of N residues, let r_α_ and r_β_ be the respective coordinates of their residues at position i, for 1, …, N. RMSD between α and β as Eq. (1):

1 $$RMSD=\sqrt{\frac{1}{N}\mathop{\sum }\limits_{i=1}^{N}{\left({r}_{\alpha ,i}-Q{r}_{\beta ,i}\right)}^{2}}$$

Where Q is the unitary rotation matrix that optimally aligns the vectors. Disulfide bonds are also considered to see if AlphaFold can correctly predict the disulfide bonds. Whatcheck^36^ and Procheck^37^ are used to assess the quality of the predicted structures. Whatcheck^36^ evaluates multiple parameters such as bond lengths, bond angles, and torsion angles of the input structure. Procheck^37^ assesses the stereochemical quality of the input structure and provides various graphical outputs. Ramachandran plot^38^ is used to evaluate the rationality of the structure, where peptide bond dihedral angles Ψ(psi) and Φ(phi) combinations are expected to located in most favored regions and allowed regions (core regions) in the plot. Ideally, a protein structure should have over 90% dihedral angles Φ-Ψ of residues in these core regions^37^.

## Data Records

**Fig. 2:**
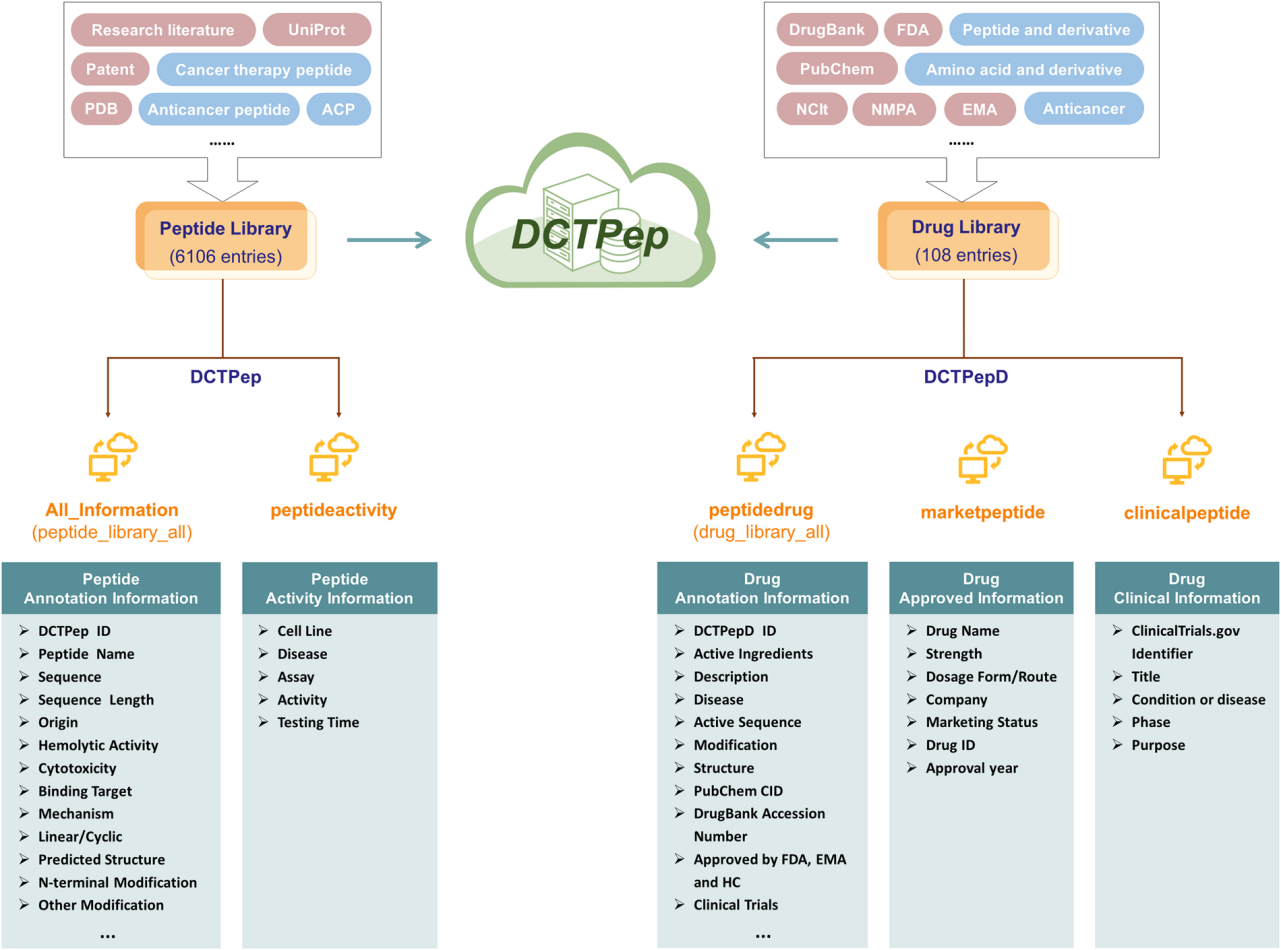
Architecture of the datasets in DCTPep.

The datasets of DCTPep are available at Figshare^39^ and contains the following files: All_information (annotation information table for storing peptide library entries), peptideactivity (activity information annotation of peptide library entries), peptidedrug (annotation information table for storing active Ingredients of drug library entries), marketpeptide (approved drug preparations information annotation of drug library entries), clinicalpeptide (clinical peptide information annotation of drug library entries), peptide_library_all (peptide library data stored in Fasta format) and prediction pdb (compressed packets for storing predicted structures). The architecture of the DCTPep is shown in Fig. 2. DCTPep contains a total of 6214 peptide entries, of which 6106 are stored in the peptide library and 108 are stored in the drug library (DCTPepD), involving over 60 targets and over 380 cancer cell lines.

Table 2 displays detailed annotation information of the data in the peptide library. Each entry in the peptide library consists of the following sections: general information, activity information, structural information, physicochemical information, literature information, and links. The peptides in the peptide library included cancer therapeutic peptides such as traditional ACP and cancer targeted peptides. Low cytotoxicity and hemolytic activity are also important criteria for developing peptide-based drugs. Therefore, in addition to anticancer activity and targets, activity information also includes cytotoxicity and hemolytic activity. All annotation information is manually extracted from the literature, and corresponding paper or patent source information is provided. The physicochemical information is calculated by Protparam and SciDBMaker^29^. For the same peptide, the emphasis of the information recorded in different databases may vary. Therefore, DCTPep provides corresponding peptide entry codes in other peptide databases.

**Table 2:** Peptide library data annotation field list.

Fields		Description
General information	DCTPep ID	The identifier of peptides in DCTPep.
	Peptide Name	Name of each peptide in DCTPep.
	Sequence	Single letter sequence of peptides.
	Sequence Length	Number of residues in the peptide sequence.
	Uniprot ID	Corresponding Uniprot link ID.
	Source	Organism source.
	Type	Native peptide or synthetic peptide.
	Classification	Various possible classifications. Including ACP, Cancer targeted peptides, Membrane-targeted mechanism…
Activity information	Anticancer activity	Anticancer activity verified by experiment (Data from literature).
	Hemolytic Activity	Hemolytic activity information against red blood cells (RBCs).
	Cytotoxicity	Cytotoxicity information against normal (non-cancerous) cell line.
	Target	The action site of peptides against cancer cell.
	Affinity	Binding affinity between peptides and targets.
	Mechanism	Mechanisms by peptides exert anticancer agents.
Structure information	PDB ID	Corresponding PDB link ID.
	Predicted Structure	Structure predicted by Alphafold.
	Helicity	Percentage of α-helix.
	Linear/Cyclic	Linear or cyclic of peptides.
	Disulfide/Other Bond	Disulfide bond (DSB) or other bond, such as N-C termini peptide bond (NCB).
	N/C-terminal Modification	The modifications of N/C-terminal according to the references.
	Other Modification	Special amino acids (out of 20 common amino acids).
	Chiral	The L/D amino acid composition of peptides.
Physicochemical Information		Formula, mass, pI, Net charge and other information, calculated by Protparam and SciDBMaker.
Literature Information		The information of peptides come from all kinds of papers or patents, and the section provides the way to find the full text.
Link		Corresponding link to other peptide databases.

The data in the drug library includes peptide drugs that have been approved or are in clinical research stage. Table 3 shows detailed annotation information for drug library data. Each entry consists of four sections: general information, structural information, external codes, and drug approval. The external codes provide identification codes for drug entries in other public databases, allowing users to obtain more comprehensive information on related entries from other sources. Approved drug formulations and clinical information can be found in the drug approval section. A total of 28 approved anticancer peptide drugs and 80 peptides in various clinical trial stages are included in the drug library.

**Table 3:** Drug library data annotation field list.

Fields		Description
General information	DCTPepD ID	Identification code for DCTPepD drug library, the field provides the unique accessing number linking to the corresponding DCTPepD entry.
	Active Ingredients	Active pharmaceutical ingredient. Substance in which the drug actually works.
	Description	Drug description. Derived from descriptions in NCI or literature sources.
	Synonyms	Other names of drug.
	Disease	Applicable diseases.
	Classification	Drug Categories.
Structure information		Molecular Formula, Molecular Weight, Active Sequence, Sequence Length, Modification, and other structure information.
External Codes		External identification code, also provides the accessing link to PubChem, DrugBank, NCI Thesaurus and GSRS.
Drug approval	Drug indication	Stemmed from DrugBank or clinical trials.
	Approved information	Approved drug formulation information, sourced from Drugs@FDA, European Medicines Agency (EMA), and Health Canada.
	Clinical information	Information sourced from ClinicalTrials.gov.

## Technical Validation

Alphafold demonstrated unprecedented accuracy in 14th Critical Assessment of protein Structure Prediction (CASP14)^34^. The study conducted by McDonald *et al*.^40^ also indicated that AlphaFold can accurately predict peptides with α-helices, β-sheets, and rich in disulfide bonds. To evaluate the accuracy of AlphaFold, 30 ACPs with experimentally determined structures were predicted by AlphaFold.

**Fig. 3:**
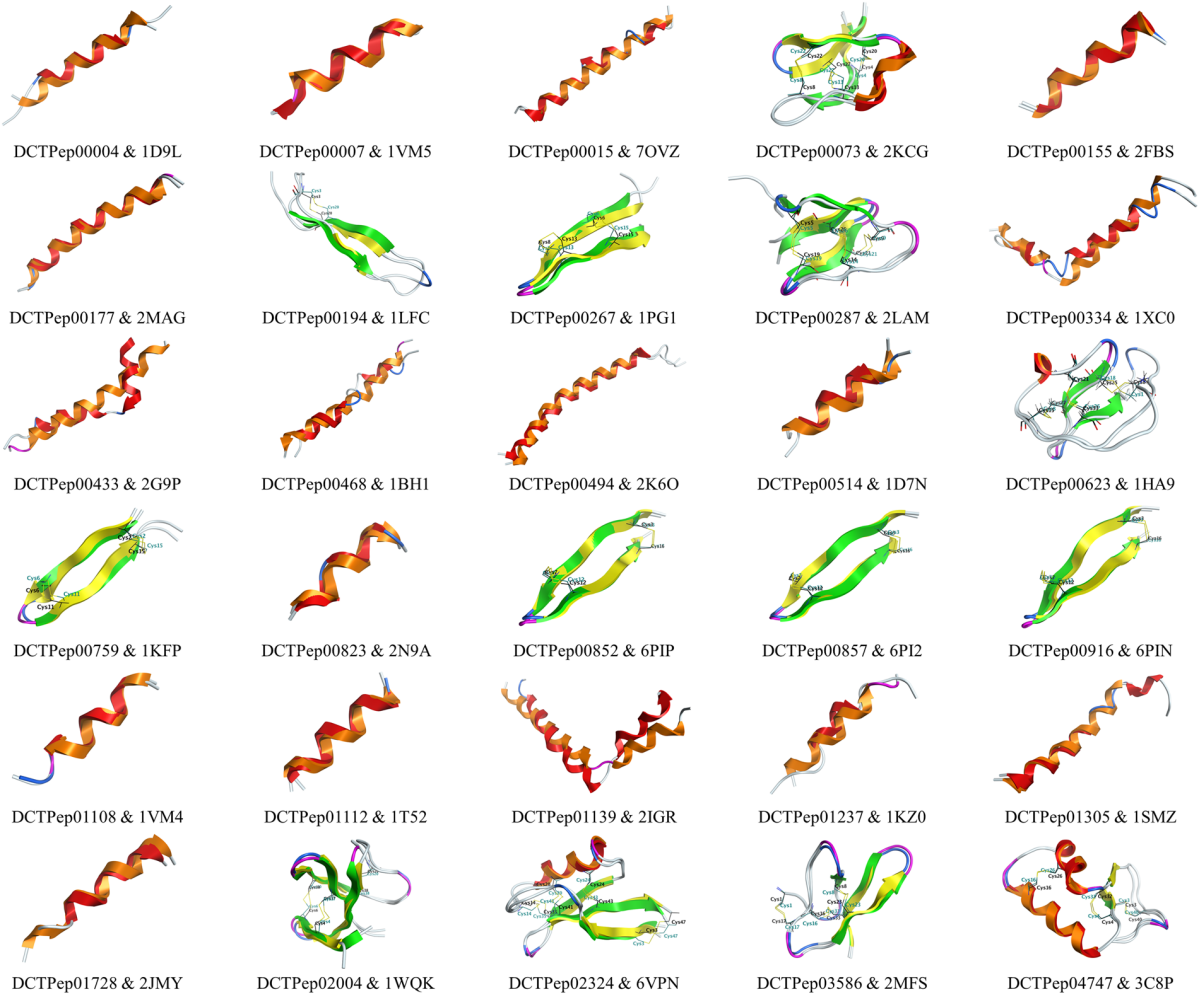
Alignment and superimposition plot of predicted structures and experimental structures. Predicted structures: helix-orange, strand-green, turn-magenta, Cys-dark cyan; Experimental structures: helix-red, strand-yellow, turn-blue, Cys-black.

Table 4 and Fig. 3 displays the comparison results between predicted structures and experimental structures, including RMSD and disulfide bond positions. The results indicate that the predicted structures have high accuracy. The deviations between the predicted and experimental structures are small, with an average of Cα (α-carbon atom) RMSD value is 1.621 Å. For structures containing disulfide bonds, AlphaFold can accurately predict the positions of the disulfide bonds. Some of the predicted structures of peptides can be directly obtained from the AlphaFold Protein Structure Database^41^, for example, AF-P82393-F1 (DCTPep00006) and AF-P80400-F1 (DCTPep00097).

**Table 4:** Comparison between predicted structures and experimental structures.

DCTPep_ID	PDB_ID	pLDDT	Cα RMSD(Å)	Disulfide bond position*
DCTPep00004	1D9L	81.43	2.314	/
DCTPep00007	1VM5	92.59	0.629	/
DCTPep00015	7OVZ	85.33	2.510	/
DCTPep00073	2KCG	89.84	0.758	TRUE
DCTPep00155	2FBS	87.92	0.419	/
DCTPep00177	2MAG	84.56	0.632	/
DCTPep00194	1LFC	76.00	2.060	TRUE
DCTPep00267	1PG1	84.8	2.145	TRUE
DCTPep00287	2LAM	84.35	2.345	TRUE
DCTPep00334	1XC0	75.86	3.583	/
DCTPep00433	2G9P	83.30	3.740	/
DCTPep00468	1BH1	79.11	3.435	/
DCTPep00494	2K6O	83.08	2.148	/
DCTPep00514	1D7N	82.51	1.927	/
DCTPep00623	1HA9	84.38	1.029	TRUE
DCTPep00759	1KFP	88.10	1.632	TRUE
DCTPep00823	2N9A	92.31	0.972	/
DCTPep00852	6PIP	91.06	0.618	TRUE
DCTPep00857	6PI2	91.14	0.688	TRUE
DCTPep00916	6PIN	90.74	0.880	TRUE
DCTPep01108	1VM4	91.98	0.598	/
DCTPep01112	1T52	82.43	0.916	/
DCTPep01139	2IGR	71.96	3.413	/
DCTPep01237	1KZ0	79.60	2.538	/
DCTPep01305	1SMZ	82.25	1.276	/
DCTPep01728	2JMY	83.34	1.985	/
DCTPep02004	1WQK	86.35	1.281	TURE
DCTPep02324	6VPN	97.15	0.878	TURE
DCTPep03586	2MFS	88.20	0.689	TURE
DCTPep04747	3C8P	95.60	0.579	TURE
Average	—	—	1.621	—

^*^“TURE” indicates consistent disulfide bond formation positions; “FALSE” indicates inconsistent disulfide bond formation positions; “/” indicates no disulfide bond formation.

**Fig. 4:**
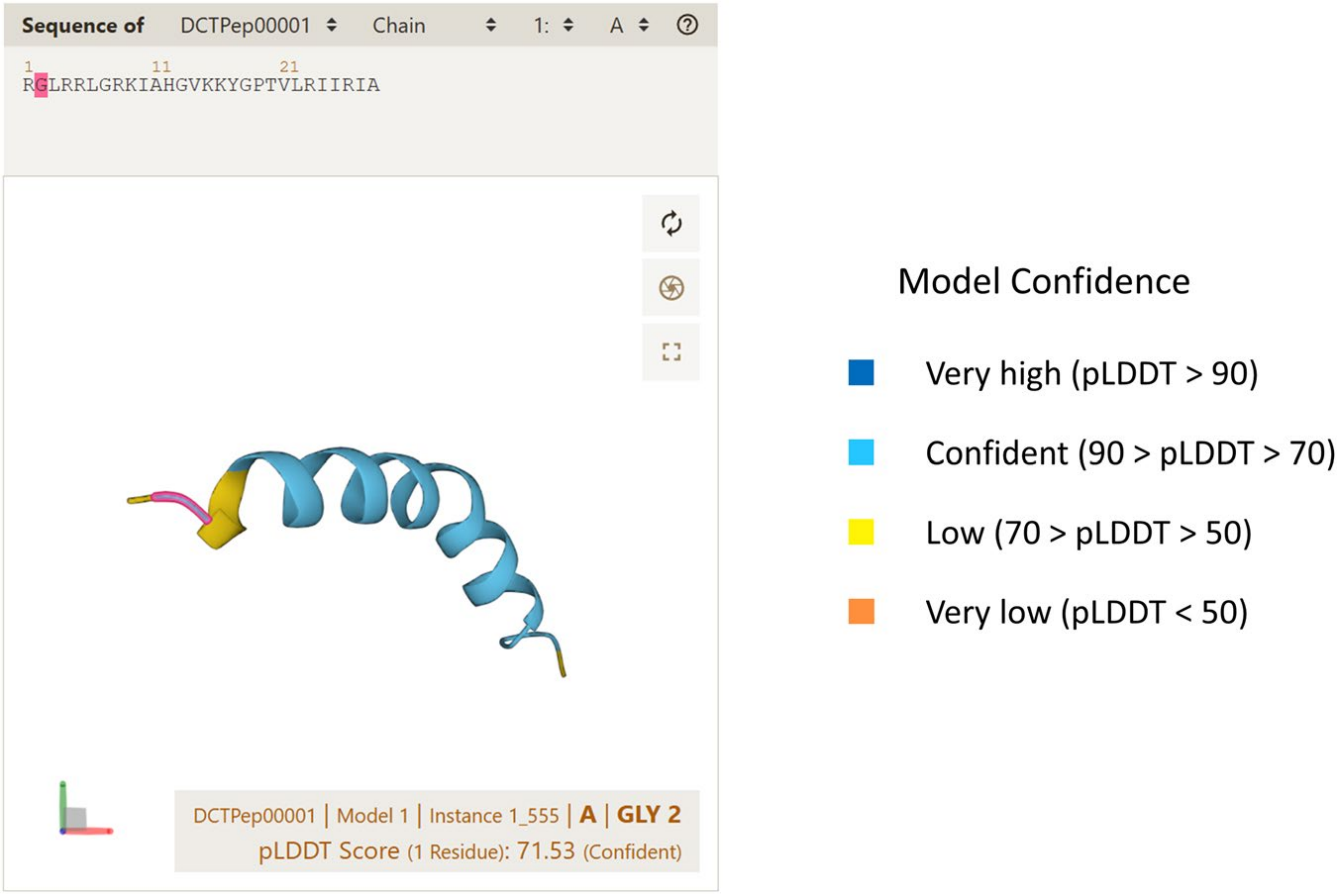
Example of the predicted structure of DCTPep00001 showing by Mol* Viewer.

pLDDT is an important parameter for assessing the confidence of predictions^34^. While using pLDDT alone to define the accuracy of predicted peptide structures may not be entirely accurate, it can still reflect their accuracy to some extent. DCTPep integrates the Mol* Viewer^42^ to display the predicted structures, where the pLDDT of each residue can be visualized in the structure^43^ (Fig. 4).

The quality assessment of the predicted structures was performed using Whatcheck^36^ and Procheck^37^ (Table 5), and the results indicate that the predicted structures are reliable. The average error rate of Whatcheck is 11.52%, which is at a relatively low level. In the Ramachandran plot generated by Procheck, the average core regions occupancy rate is 95.11%, only the DCTPep00623 has a low occupancy rate of core regions. The average disallowed regions occupancy rate is 0.26%, only DCTPep00267 has one residue present in the disallowed regions. These errors are within an acceptable range.

**Table 5:** The results of predicted structures in Whatcheck and Procheck.

DCTPep_ID	Whatcheck			Procheck		
	Total metrics	Error	Error rate	Core regions	Additional/generous allowed regions	Disallowed regions
DCTPep00004	40	4	10.00%	100.00%	0.00%	0.00%
DCTPep00007	40	4	10.00%	100.00%	0.00%	0.00%
DCTPep00015	42	4	9.52%	100.00%	0.00%	0.00%
DCTPep00073	42	5	11.90%	87.50%	12.50%	0.00%
DCTPep00155	42	4	9.52%	100.00%	0.00%	0.00%
DCTPep00177	42	4	9.52%	100.00%	0.00%	0.00%
DCTPep00194	43	6	13.95%	100.00%	0.00%	0.00%
DCTPep00267	40	5	12.50%	92.30%	0.00%	7.70%
DCTPep00287	44	5	11.36%	81.80%	18.20%	0.00%
DCTPep00334	43	4	9.30%	96.20%	3.80%	0.00%
DCTPep00433	43	4	9.30%	100.00%	0.00%	0.00%
DCTPep00468	42	4	9.52%	100.00%	0.00%	0.00%
DCTPep00494	45	5	11.11%	90.60%	9.40%	0.00%
DCTPep00514	38	4	10.53%	100.00%	0.00%	0.00%
DCTPep00623	42	6	14.29%	69.20%	30.80%	0.00%
DCTPep00759	41	5	12.20%	86.70%	13.30%	0.00%
DCTPep00823	39	4	10.26%	100.00%	0.00%	0.00%
DCTPep00852	40	6	15.00%	92.90%	7.10%	0.00%
DCTPep00857	40	5	12.50%	92.90%	7.10%	0.00%
DCTPep00916	40	4	10.00%	92.90%	7.10%	0.00%
DCTPep01108	40	4	10.00%	100.00%	0.00%	0.00%
DCTPep01112	38	4	10.53%	100.00%	0.00%	0.00%
DCTPep01139	40	6	15.00%	100.00%	0.00%	0.00%
DCTPep01237	39	4	10.26%	100.00%	0.00%	0.00%
DCTPep01305	39	4	10.26%	100.00%	0.00%	0.00%
DCTPep01728	39	5	12.82%	100.00%	0.00%	0.00%
DCTPep02004	45	6	13.33%	90.30%	9.70%	0.00%
DCTPep02324	45	6	13.33%	95.10%	4.90%	0.00%
DCTPep03586	43	7	16.28%	87.50%	8.30%	0.00%
DCTPep04747	43	5	11.63%	97.40%	2.60%	0.00%
Average	—	—	11.52%	95.11%	4.49%	0.26%

## Data Availability

DCTPep can be freely accessed at http://dctpep.cpu-bioinfor.org/. The data are stored in the Figshare repository available at 10.6084/m9.figshare.25796353.v1.
